# Supplementary material for: Incidence of Differentiation Syndrome Associated with Treatment Regimens in Acute Myeloid Leukemia: A Systematic Review of the Literature
Source: J Clin Med. 2020 Oct 18;9(10):3342. doi: 10.3390/jcm9103342 (PMC7603213; doi:10.3390/jcm9103342)
Supplement: Supplementary file 1 [file jcm-09-03342-s001.pdf]

Table S1. Complete data extraction of included clinical trials (n = 39) and patient characteristics, treatment regimens, and outcomes

CLINICAL TRIALS			PATIENTS					TREATMENT				Outcomes					
**reference**	**trial type**	**duration**	**total**	**age**	**(range)**	**male**	**female**	**criterion**	**induction regimen**			**# DS**	**% DS**	**deaths**	**%**	**TTO [d]**	**(range)**
Zhu, 2013	non-inferiority	11/07-09/11	231	37	(15-60)	126	105					51	22.1	0	0		
			117	39	(15-60)	65	52	randomised	ATRA 25 mg/m2	ATO 0.16 mg/kg		29	24.8	0	0		
			114	33	(15-60)	61	53	randomised	ATRA 25 mg/m2	RIF 60 mg/kg		22	19.3	0	0		
Iland, 2012	phase 2	11/04-11/09	124	44	(3-78)	62	62					17	13.7	0	0		
			108					≤ 60 y.	ATRA 45 mg/m2/d tid	ATO 0.15 mg/kg/d qd d9-36	idarubicin 12 mg/m2/d d2,4,6,8						
			9					61 - 70 y.	ATRA 45 mg/m2/d tid	ATO 0.15 mg/kg/d qd d9-36	idarubicin 9 mg/m2/d d2,4,6,8						
			7					> 70 y.	ATRA 45 mg/m2/d tid	ATO 0.15 mg/kg/d qd d9-36	idarubicin 6 mg/m2/d d2,4,6,8						
Abaza, 2017; Ravandi, 2009	non-randomised	07/02-05/15	187	50	(14-84)	97	90					21	11.2	0	0		
								WBC < 10*10^9/l old	ATRA 45 mg/m2/d bid	ATO 0.15 mg/kg/d qd from d10							
								WBC ≥ 10*10^9/l old	ATRA 45 mg/m2/d bid	ATO 0.15 mg/kg/d qd from d10	gemtuzumab ozogamicin 9 mg/m2/d qd d1						
								WBC < 10*10^9/l new	ATRA 45 mg/m2/d bid	ATO 0.15 mg/kg/d qd from d1							
								WBC ≥ 10*10^9/l new	ATRA 45 mg/m2/d bid	ATO 0.15 mg/kg/d qd from d1	gemtuzumab ozogamicin 9 mg/m2/d qd d1						

Dai, 2009	comparative	03/03-03/08	162			93	69					5	3.1	0	0
			72	34	(14-69)	39	33		ATRA 45 mg/m2/d			2	2.8		
			90	32	(14-67)	54	36		ATRA 45 mg/m2/d	ATO 10 mg/d d1-28		3	3.3		
Platzbecker, 2016; LoCoco, 2013	phase 3 noninferiority	10/07-01/13	266			130	136					38	14.3	2	0.8
			129	46.6	(19-70)	60	69	randomised	ATRA 45 mg/m2/d bid	ATO 0.15 mg/kg/d qd		21	16.3	0	0
			137	46.6	(18-70)	70	67	randomised	ATRA 45 mg/m2/d bid	idarubicin 12 mg/m2/d qd d2,4,6,8		17	12.4	2	1.5
Yang, 2018	comparative	09/11-01/17	82	9.4	(1-16)	51	31					5	6.1		
			42	7.8	(1-13)	29	13	randomised	ATRA 25 mg/m2/d	mitoxantrone 10 or 7 mg/m2/d d3 or d2-4	ATO 0.16 mg/kg/d qd from d5/6	4	9.5		
			40	9.9	(2-16)	22	18	randomised	ATRA 25 mg/m2/d	mitoxantrone 10 or 7 mg/m2/d d3 or d2-4	RIF 135 mg/kg/d tid from d5/6	1	2.5		
Burnett, 2015	phase 3	04/09-10/13	235	47	(16-77)	120	115					55	23.4	0	0
			119	47	(16-77)	60	59	randomised	ATRA 45 mg/m2/d bid	idarubicin 12 mg/m2/d qd d2,4,6,8		25	21.0		
			116	47	(16-75)	60	56	randomised	ATRA 45 mg/m2/d bid	ATO 0.3 mg/kg/d qd d1-5 & wk2-8		30	25.9		

Imaizumi, 2010	pediatric	08/97-03/04	58	11	(1-16)	31	27		ATRA 45 mg/m2/d	daunorubicin 45 mg/m2/d d6-8	cytarabine 200 mg/m2/d d6-12		4	7.3	0	0		
Sanz, 2010	phase 4	07/05-04/09	402	42	(3-83)	209	193						106	28.5	4	1.1		
								20-70 y.	ATRA 45 mg/m2/d bid	idarubicin 12 mg/m2/d qd d2,4,6,8								
			22					> 70 y.	ATRA 45 mg/m2/d bid	idarubicin 12 mg/m2/d qd d2,4,6								
								< 20 y.	ATRA 25 mg/m2/d bid	idarubicin 12 mg/m2/d qd d2,4,6,8								
Lengfelder, 2009	single-arm	12/94-12/05	142	40	(16-60)	59	83	1st induction cycle	ATRA 45 mg/m2/d	6-thioguanine 100mg/m2 bid d3-9	cytarabine 100 mg/m2 d1-2, bid d3-8	daunorubicin 60 mg/m2 d3-5	28	21.1	1	0.8		
			131					2nd induction cycle	cytarabine 3g/m2 bid d21-23	mitoxantrone 10 mg/m2 d23-25								
De Botton, 2003	comparative	04/93-10/98	306			135	171						39	12.7	4	1.3		
			122	45.5	(35-54)	56	66	randomised	ATRA 45 mg/m2/d				22	18.0	3	2.5	10	
			184	45	(34-55)	79	105	randomised	ATRA 45 mg/m2/d	daunorubicin 60 mg/kg/d d3-5	cytarabine 200 mg/m2/d d3-9		17	9.2	1	0.5	10.5	
Montesinos, 2009	comparative	11/96-06/05	739	40	(2-83)	374	365						183	24.8	10	1.4	12	(0-46)
								20-70 y.	ATRA 45 mg/m2/d bid	idarubicin 12 mg/m2/d qd d2,4,6,8								

								> 70 y.	ATRA 45 mg/m2/d bid	idarubicin 12 mg/m2/d qd d2,4,6							
								< 20 y.	ATRA 25 mg/m2/d bid	idarubicin 12 mg/m2/d qd d2,4,6,8							
LoCoco, 2010; Testi 2005	phase 2	04/93-05/00	752			404	348					84	11.3	1	0.1		
			642	38.2	(18-61)	349	293	≥ 18 y.	ATRA 45 mg/m2/d	idarubicin 12 mg/m2/d qd d2,4,6,8		82	12.9	1	0.2		
			110	11.6	(1-18)	55	55	< 18 y.	ATRA 25 mg/m2/d	idarubicin 12 mg/m2/d qd d2,4,6,8		2	1.8	0	0	7.5	(4-11)
LoCoco, 2010	phase 2	06/00-10/06	453	40.9	(18-61)	229	224		ATRA 45 mg/m2/d	idarubicin 12 mg/m2/d qd d2,4,6,8		46	10.3	1	0.2		
Colovic, 1997	comparative	02/92-11/96	30			12	18					4	13.3	4	13.3		
			15	40	(16-65)	4	11	WBC < 5*10^9/l	ATRA 45 mg/m2/d bid			1	6.7	1	6.7		
			15	40	(18-60)	8	7	WBC > 5*10^9/l	ATRA 45 mg/m2/d bid	daunorubicin 50 mg/m2/d 3d	cytarabine 200 mg/m2/d 7d	3	20.0	3	20		
Fenaux, 1993	comparative	03/91-12/92	101	40	(6-67)	53	48										
			54	41.5	(6-63)	30	24	randomised	ATRA 45 mg/m2/d	daunorubicin 60	cytarabine 200	3	5.6	0	0	20	(14-24)

										mg/m2/d<br>3d	mg/m2/d<br>7d							
			47	40	(17-67)	23	24	randomised		daunorub<br>icin<br>60<br>mg/m2/d<br>2x3d	cytarabi<br>ne<br>200<br>mg/m2/d<br>2x7d							
Powell,<br>2010	phase 3	06/99-<br>03/05	48<br>1		(15-79)	247	234		ATRA<br>45<br>mg/m2/d<br>bid	daunorub<br>icin<br>50<br>mg/m2/d<br>qd d3-6	cytarabi<br>ne<br>200<br>mg/m2/d<br>d3-9		177	36.8				
Frankel,<br>1994	single-arm	06/90-<br>06/92	56		(9-75)	25	31		ATRA<br>45<br>mg/m2/d<br>bid				13	23.2	5	8.9		
Tallman,<br>1997;<br>Tallman,<br>2000	comparative	04/92-<br>02/95	34<br>6			179	167											
			17<br>2	37	(1-81)	82	90	randomised	ATRA<br>45<br>mg/m2/d<br>bid				44	26.3	2	1.2	11	(2-47)
			17<br>4	38	(1-74)	97	77	randomised	daunorubic<br>in<br>45<br>mg/m2/d<br>qd d1-36	cytarabine<br>100<br>mg/m2/d<br>d1-7								
Castaigne,<br>1993	single-arm	05/90-<br>01/92	30	56	(10-81)	13	17		ATRA<br>25<br>mg/m2/d<br>bid				2	6.7	2	6.7		
Douer,<br>2001	single-arm	03/93-<br>05/99	69	44	(5-82)	40	29		liposomal<br>ATRA<br>90 mg/m2<br>qad				18	26.1	1	1.4		
Advani,<br>1999	comparative	02/92-<br>12/96	43		(7-60)	28	15		ATRA<br>45<br>mg/m2/d				14	32.6	6	14.<br>0	10	(4-26)
de<br>Medeiros,<br>1998	single-arm	03/92-<br>11/95	37	17.<br>5	(9-69)	17	20		ATRA<br>45<br>mg/m2/d				4	10.8	0	0		
Mandegar<br>y, 2010	single-arm	01/06-<br>01/07	20	31	(15-62)	7	13		ATO<br>15 mg/kg/d<br>qd				12	60.0	1	5.0		

Ghavamzadeh, 2006	single-arm	05/00-01/05	111	27	(6-79)	51	60		ATO 15 mg/kg/d qd		23	20.7	8	7.2	
Mathews, 2006	phase 4	01/98-12/04	72	28	(3-75)	38	34				5	6.9	0	0	13.2*	(6-21)
								adults	ATO 10 mg/d	hydroxyurea 0-4 g/d						
								pediatric patients	ATO 0.15 mg/kg/d	hydroxyurea 0-30 mg/kg/d qd-qid						
Zhou, 2010	single-arm	08/02-01/07	19	10	(4-15)	11	8				2	10.5	0	0		
			5					4-6y.	ATO 0.2 mg/kg/d qd							
			14					> 6y.	ATO 0.16 mg/kg/d qd							
Hao, 2013	single-arm	02/00-04/10	46	8	(mean)	35	11		ATO 0.17-0.33 mg/kg/d qd		8	17.4				
Soignet, 1998	single-arm		12	33.5	(9-75)				ATO 0.06-0.2 mg/kg/d		2	16.7	0	0		
Soignet, 2001	single-arm	04/98-04/99	40			24	16		ATO 0.15 mg/kg/d		10	25.0	0	0		
Jin, 2006	single-arm	09/01-12/04	30		(18-65)	18	12		ATO 10 mg qd		9	30.0	0	0	13.9*	(5-25)
Shigeno, 2005; Onishi, 2002	phase 2	03/99-08/04	34	47	(17-82)	22	12		ATO 0.15 mg/kg/d		8	23.5	0	0		
DiNardo, 2018	phase 1	03/14-05/17	258	68	(18-89)	137	121	dose-escalation	ivosidenib 100 mg bid/300-1200 mg qd		29	11.2	0	0		
Stein, 2017; Fathi, 2018	phase 1	09/13-04/16	239	70	(19-100)	137	102	dose-escalation	enasidenib 30-150 mg bid/50-650 mg qd		23	9.6			48	(10-340)

Tsai, 2008	phase 1		27	69	(51-82)	19	8	dose-escalation	bexarotene 100-300 mg/m2, 400 mg/m2			2	7.4	0	0		
Tobita, 1997	single-arm	03/95-04/96	24	49	(19-76)	13	11		tamibarotene 6 mg/m2/d bid			1	4.2	0	0	18	
Zhang, 2013	non-randomised	03/96-12/02	33	65	(60-79)							5	15.2	0	0		
								WBC ≤ 20*10^9/l	ATO 0.16 mg/kg/d qd								
								WBC > 20*10^9/l	ATO 0.08 mg/kg/d qd	daunorubicin 40 mg d1-3	cytarabine 50-100 mg d1-5						
Asou, 2007	comparative	05/97-06/02	283	48	(15-70)	158	125					60	21.2	2	0.7		
			85					WBC < 3*10^9/l	ATRA 45 mg/m2/d tid								
			139					3*10^9/l ≤ WBC < 10*10^9/l	ATRA 45 mg/m2/d tid	idarubicin 12 mg/m2/d qd d1-2	cytarabine 80 mg/m2/d d1-5						
			52					WBC > 10*10^9/l	ATRA 45 mg/m2/d tid	idarubicin 12 mg/m2/d qd d1-3	cytarabine 100 mg/m2/d d1-5						
Asou, 2001	non-randomised	01/92-05/97	369	46	(15-85)	173	196					28	7.6	1	0.3		
			126					WBC < 3x10^9/l	ATRA 45 mg/m2 tid								
			243					WBC ≥ 3x10^9/l	ATRA 45 mg/m2 tid	daunorubicin 40 mg/m2/d qd 3d	enocitabine 200 mg/m2/d qd 5d						

Abbreviations: DS = Differentiation syndrome; TTO = Time to treatment onset, ATRA : All trans retinoic acid, ATO = arsenic trioxide, mg = milligrams, m^2 = meters squared, d= day, tid kg = kilograms, qd = once daily, bid = twice daily, tid = three times daily, qid = four times daily, qad = ever other day; d5= day 5 after start of treatment WBC = white blood cells
Variables: total = total number of patients in clinical trial (arm); age = median age in years; allocation = criterion for the assignment of patients to the respective clinical trial arm; Primary treatment = describes drugs, doses and frequencies of administration of the drugs administered for remission induction; %DS = proportion of patients who experienced DS in

the respective clinical trial (arm); % deaths = proportion of patients who died as a consequence of DS; TTO = median (mean if marked with *) time to onset of DS after treatment start given in days

Notes: Information applicable to the whole clinical trial population is highlighted in grey. In the rows underneath, the individual trial arms are described (one arm per row). In the induction regimen description the dosing information is provided beneath the corresponding drug name. If a field is empty in the table, the value was not stated in the publication.
